# Hilar/mediastinal and cutaneous drug-induced sarcoidosis-like reaction associated with immune checkpoint inhibitors in metastatic colorectal cancer: a case report

**DOI:** 10.3389/fimmu.2023.1203621

**Published:** 2023-07-10

**Authors:** Tamotsu Sagawa, Yasushi Sato, Hiroyuki Nagashima, Kohichi Takada, Mamoru Takahashi, Masahiro Hirakawa, Kyoko Hamaguchi, Fumito Tamura, Koshi Fujikawa, Koichi Okamoto, Yutaka Kawano, Masahiro Sogabe, Hiroshi Miyamoto, Tetsuji Takayama

**Affiliations:** ^1^ Department of Gastroenterology, Hokkaido Cancer Center, Sapporo, Hokkaido, Japan; ^2^ Department of Gastroenterology and Oncology, Tokushima University Graduate School of Biomedical Sciences, Tokushima, Japan; ^3^ Department of Medical Oncology, Sapporo Medical University School of Medicine, Sapporo, Hokkaido, Japan; ^4^ Department of Respiratory Medicine and Allergology, Sapporo Medical University School of Medicine, Sapporo, Hokkaido, Japan

**Keywords:** immune checkpoint inhibitor, colorectal cancer, sarcoidosis, microsatellite instability, side effects

## Abstract

**Background:**

Immune checkpoint inhibitors (ICIs) are the standard treatment for metastatic colorectal cancer (mCRC) with high microsatellite instability (MSI-H). Among immune-related adverse events (irAEs), drug-induced sarcoidosis-like reactions (DISR) are often difficult to differentiate from cancer progression.

**Main Body:**

This is a case of a woman in her mid-60s, with mCRC (RAS wild/BRAF mutant/MSI-H) and abdominal lymph node metastasis, treated with four courses of ipilimumab + nivolumab every 3 weeks, followed by nivolumab every 2 weeks as third-line treatment. After treatment, the original lymph node metastases shrank, but hilar/mediastinal lymph nodes appeared. Endoscopic ultrasound-guided fine-needle aspiration of these lymph nodes revealed multiple epithelioid granulomas without necrosis, indicating a sarcoidosis-like reaction. Fluorodeoxyglucose-positron emission tomography scan showed abnormal subcutaneous accumulation in bilateral forearms and bilateral knee joints. Biopsy of the cutaneous lesions was also performed, which revealed epithelioid granulomas. As the patient had no symptoms in other organs, no specific therapeutic intervention was administered. After the discontinuation of immunotherapy, the sarcoidosis-like reaction regressed without cancer relapse.

**Conclusions:**

Clinicians should be aware of the possibility of DISR as an irAE during the ICI treatment of mCRC. In suspected cases of DISR following ICI therapy, it is important to differentiate between cancer progression and DISR through histological diagnosis for the subsequent strategy, as radiological and serological findings are not definitive.

## Introduction

In recent years, the use of immune checkpoint inhibitors (ICIs) has revolutionized cancer treatment, owing to its potential for significant long-term survival benefits in some cancers. In metastatic colorectal cancer (mCRC), ICIs have demonstrated their efficacy in the treatment of mismatch-repair-deficient (dMMR) tumors or high microsatellite instability (MSI-H) tumors (1). However, along with robust antitumor activity, ICI therapy also has nonspecific systemic effects in the setting of immune activation. Toxicity manifests as a wide range of immune-related adverse events (irAEs), including dermatitis, endocrine disorders, autoimmune proctitis, pneumonia, hepatitis, and neuropathy (2).

Drug-induced sarcoidosis-like reaction (DISR) is a granulomatous disease whose features are indistinguishable from sarcoidosis, with both disease entities having similar histology, organ involvement patterns, and clinical manifestations (3). The drugs reported to be associated with DISR include interferon-α, interleukin 4/13 blocker, highly active antiretroviral therapy, and tumor necrosis factor-α antagonists. However, ICI therapy has recently been reported to cause DISR, which is recognized as a rare but important irAE (4). Although DISR has not yet been reported in the treatment of colorectal cancer, the incidence of radiological diagnoses of DISR in anti-CTLA-4 treated cases has been reported to be 5% to 6.7% and 0.2% for anti-PD-1/PD-L1 blockade in melanoma, lung cancer, and other cancers (5, 6).

Because ICI therapy has recently played a major role in the treatment of colorectal cancer in MSI-H cases, it is necessary to understand the characteristics of DISR before the further use of ICI therapy in the treatment of colorectal cancer. This is because DISR can be misinterpreted in imaging studies as an indication of treatment failure or tumor progression. Biopsy of suspected tissues should be considered to distinguish DISR from tumor progression (4).

Herein, we report a case of nivolumab plus ipilimumab treatment for MSI-H colorectal cancer that resulted in partial response but developed hilar/mediastinal and cutaneous DISR that mimicked disease progression during treatment. We performed endoscopic ultrasound-guided fine needle aspiration (EUS-FNA) of the mediastinal lymph nodes and were able to differentiate between sarcoid reactions and metastatic lymph nodes. To the best of our knowledge, this is the first case of DISR attributed to ICI in mCRC to be reported in the literature.

## Case presentation

**Figure 1:**
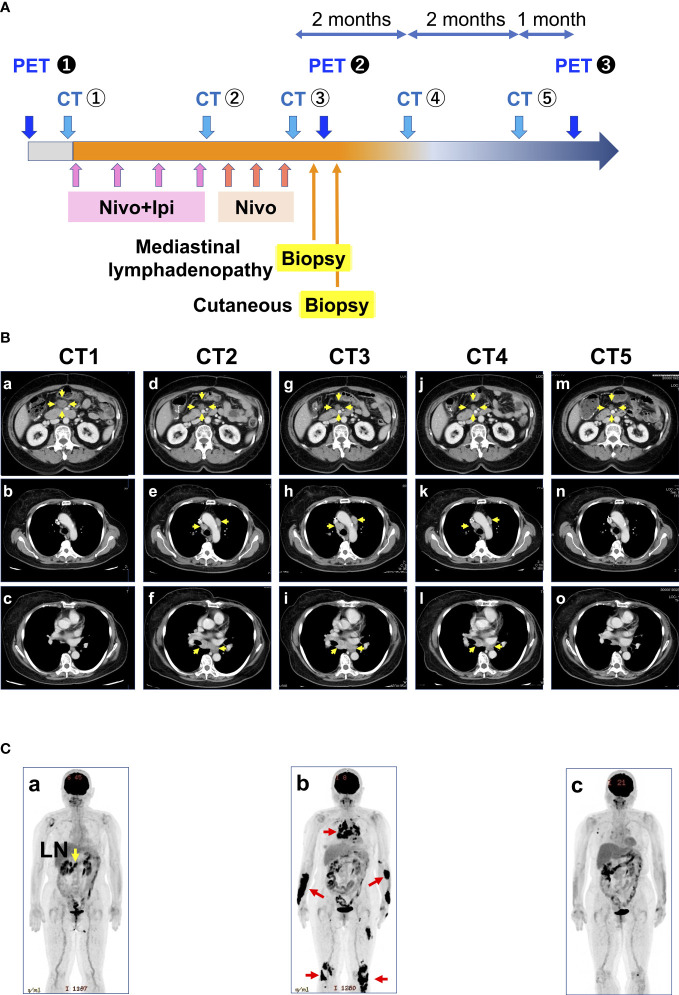
Treatment course of the patient. **(A)** History of treatment and examination details. **(B)** Representative CT images taken during the course of the disease: (a-c) correspond to CT1 of A; (d-f) correspond to CT2 of A; (g-i) correspond to CT3 of A; (j-l) correspond to CT4 of A; and (m-o) correspond to CT5 of A Yellow arrows indicate metastases. **(C)** Representative PET images during the course of the disease correspond to PET1 of A. (b) Corresponds to PET2 of A. Red arrows indicate sarcoidosis-like reactions. (c) corresponds to PET3 of A. CT, computed tomography; PET, positron emission tomography; LN, lymph node metastasis.

**Figure 2:**
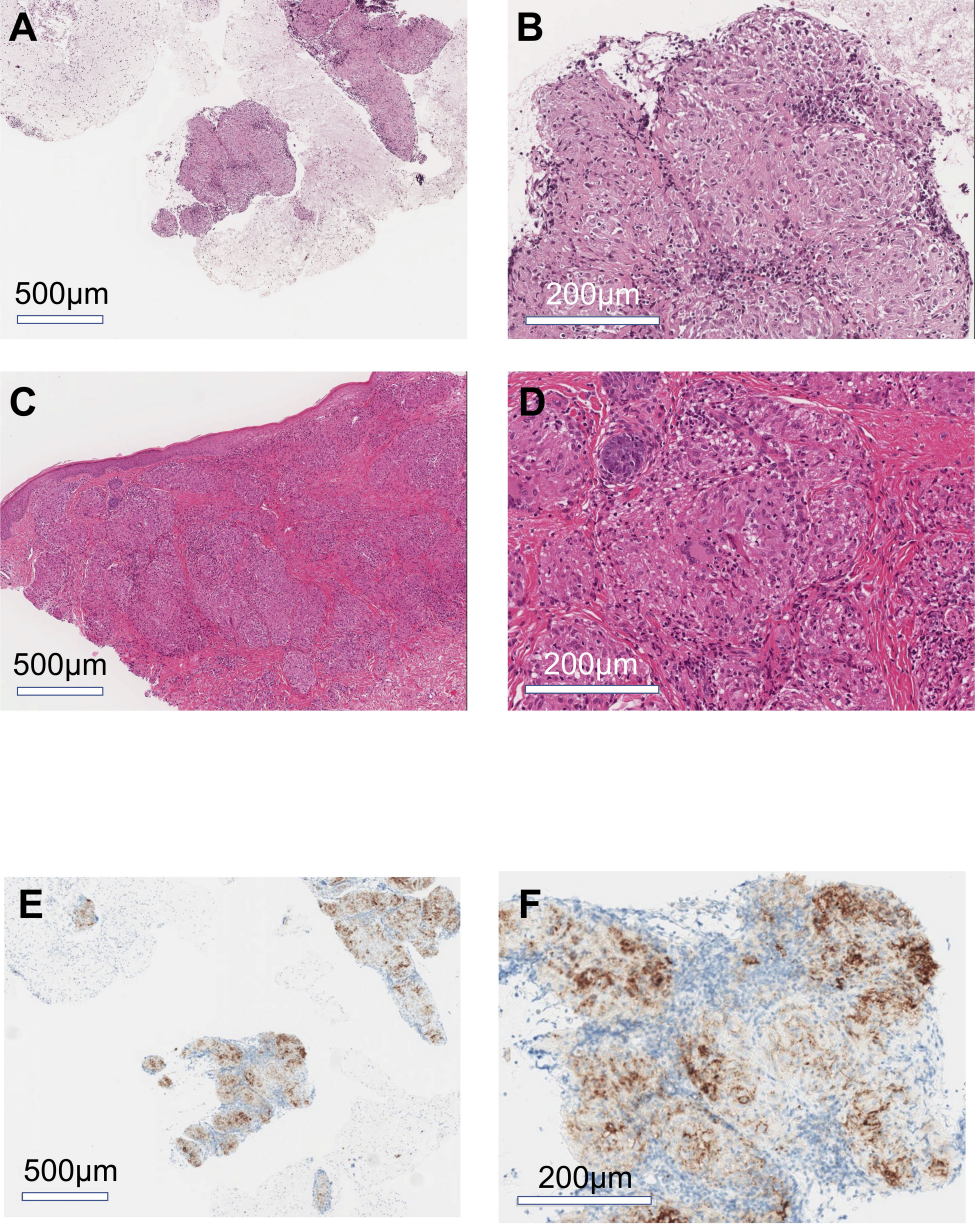
Histology of mediastinal lymphadenopathy and cutaneous lesions. Representative images of hematoxylin-eosin stain of mediastinal lymph node **(A, B)** and cutaneous lesions **(C, D)**. Representative images of immunohistochemical staining for PDL1 in the mediastinal lymph nodes **(E, F)**. **(A, C, E)** low magnification images; **(B, D, F)** high magnification images.

A woman in her mid-60s diagnosed with ascending colon cancer (Stage IV, RAS wild/BRAF mutant/MSI-H) underwent right hemicolectomy, partial resection of the small intestine, and gastric jejunal bypass (R1 resection), followed by modified fluorouracil, leucovorin, and oxaliplatin (mFOLFOX6) +bevacizumab for 17 months. The best response was stable disease. As a second-line treatment, cetuximab and encorafenib were administered for 3 months, but the best response was progressive disease due to enlargement of the abdominal lymph nodes. Hence, the patient was treated with ipilimumab (1 mg/kg) and nivolumab (240 mg) as a third-line treatment (**Figure 1A**). Computed tomography scan after four courses of ipilimumab and nivolumab followed by three courses of nivolumab treatment (12 weeks after starting ICI therapy) revealed hilar/mediastinal lymphadenopathies, whereas abdominal lymph node metastasis markedly regressed (**Figures 1B, C**). Hilar/mediastinal lymph node enlargement was suspected to be a new metastatic lesion in colorectal cancer. However, because the patient had a history of breast cancer, the possibility of metastatic recurrent lesions from breast cancer was also considered. The patient underwent EUS-FNA for the histologic diagnosis of mediastinal lymphadenopathies, which revealed epithelioid cell granuloma (**Figures 2A, B**). Fluorodeoxyglucose (FDG)-positron emission tomography (PET) scan revealed FDG uptake in the hilar/mediastinal lymphadenopathy and cutaneous lesions of the extremities (**Figure 1C**). Cutaneous biopsy also revealed epithelioid cell granulomas (**Figures 2C, D**). Angiotensin- converting enzyme (ACE) (28.7 U/l [7-25.0U/l]) and soluble interleukin-2 receptor (sIL2R) (1181 U/ml [122-496 U/ml]) were slightly increased. Based on these findings, we diagnosed the patient with DISR secondary to ICIs. As she had no central nervous system, ocular, cardiac, or other organ system symptoms, no specific therapeutic interventions were given. After discontinuation of immunotherapy, the sarcoidosis-like reaction regressed without cancer relapse (**Figures 1B, C**). As of the writing of this report, the patient had maintained tumor regression for 1.5 years after starting ICI therapy.

## Discussion

Herein, we report a case of mCRC in which hilar/mediastinal and cutaneous DISR developed during ICI treatment. Because the exact pathophysiology of DISR/sarcoidosis remains unknown, we can only speculate whether ICI therapy induces DISR or whether it induces sarcoidosis. This case was diagnosed as highly likely DISR according to the diagnostic criteria of Chopra et al. (7) based on the findings that there was no alternate cause of granulomatous inflammation, and improvement was observed after the discontinuation of ICI therapy.

Although the pathophysiology of DISR is not yet fully understood, several possible mechanisms have been postulated for different drug classes (3, 7). Sarcoidosis has historically been considered a T-helper (Th) 1 disease which is contributing to granuloma formation. Recent data demonstrate that T-helper (Th)17.1 cells, a specific subset of CD4+ T cells producing interferon-γ and interleukin-17, which exhibit high pathogenicity in inflammatory diseases, seem to play a crucial role in the development of sarcoidosis and regulatory T cell (Treg) dysfunction (8). In addition, PD-1 blockade in cancer patients increases the production of Th1/Th17 cytokines in response to antigens (9). Thus, it is possible that ICI-related Th1/Th17 polarization is a part of DISR pathophysiology and resembles the T-cell subset changes found in sarcoidosis (8). Another proposed mechanism for ICI-induced sarcoidosis-like reactions involves an increase in the number and function of Th17 cells, which has been observed in patients who received anti–CTLA-4 treatment (10). These reports suggest that ICI therapy amplifies the effects of Th17.1 cells thus causing DISR/sarcoidosis.

Braun et al. have reported an increased number of PD-1+ CD4+ T cell alveolar lavages in patients with pulmonary sarcoidosis (11). Additionally, PD- L1 has been shown to be upregulated in sarcoid lung granulomas but not in healthy lung tissue. Furthermore, the blockade of the PD-1 pathway restored the proliferative capacity of sarcoidosis CD4+ T cells to levels consistent with those of healthy control subjects (9). Interestingly, the sarcoidotic granulomas in the current case showed PD-L1 membrane staining (**Figures 2E, F**). These findings suggest that the association between PD-1 and PD-L1 has immunological consequences in the pathogenesis of DISR or sarcoidosis.

In the case presented, the mediastinal lymph nodes, hilar lymph nodes, and skin were the main sites of involvement, which is consistent with previous reports (4). In most patients, as in the present case, the disease is noted in the first imaging study per protocol, and the median onset of DISR after ICI initiation is 14 weeks (4). However, even with these identified characteristics of DISR, it is difficult to distinguish DISR from tumor progression using radiographic images or serum markers. This is a major diagnostic challenge for DISR, and misdiagnosis of DISR as tumor progression may affect the decision to continue treatment. Therefore, DISR should be accurately recognized during ICI treatment. When DISR is suspected during treatment, a biopsy is considered necessary. In the current case, EUS-FNA was performed for the differential diagnosis of mediastinal portal lymph node/mediastinal lymph nodes, resulting in a definitive diagnosis.

The DISR treatment is similar to that of systemic sarcoidosis. In most cases of ICI treatment, DISR has been reported to improve after discontinuation of ICI (4). As in this case, follow-up with cessation of the responsible drugs is considered enough if there are no symptoms or involvement of organs, including the central nervous system, eye, and heart.

Recently, the relationship between irAEs and antitumor responses in cancer patients treated with ICI has been of interest (12). Patients with advanced metastatic disease who develop DISR seem to have favorable outcomes, or at least comparable outcomes to those of patients without DISR (13, 14). The patient in this case remained untreated for 1.5 years after discontinuation of ICI, but no signs of recurrence have been observed despite the BRAF mutation in mCRC, suggesting an association between irAEs and treatment response.

## Conclusion

Clinicians should be aware of the possibility of DISR as an irAE during ICI therapy for mCRC. In suspected cases of DISR following ICI therapy, the differential diagnosis of cancer progression is crucial for subsequent strategies. Histological examination should be considered because enlarged lymph nodes and other symptoms may be misinterpreted as an indication of the progression of the underlying cancer.

## Data availability statement

The original contributions presented in the study are included in the article/supplementary material. Further inquiries can be directed to the corresponding author.

## Ethics statement

The studies involving human participants were reviewed and approved by Ethics Committee of Hokkaido Cancer Center. The patients/participants provided their written informed consent to participate in this study. Written informed consent was obtained from the individual(s) for the publication of any potentially identifiable images or data included in this article. Written informed consent was obtained from the participant/patient(s) for the publication of this case report.

## Author contributions

TS, YS did literature research and wrote the manuscript; TS treated the patient; HN, KT, MT, MH, KH, FT, and KF were part of the management team for the patients. KO, YK, MS, HM, and TT supervised the treatment and were involved in data analysis. All authors contributed to the article and approved the submitted version. TS and YS contributed equally.
